# Local Ablative Treatment of the Primary Tumour in Patients With Metastatic Breast Cancer: A Retrospective Observational Study

**DOI:** 10.7759/cureus.84020

**Published:** 2025-05-13

**Authors:** Maria Marín Alcalá, Marta Andrés Granyó, Dánae Guerra Fernández, Jairo Cortés Prados, Esther Rubio Calatayud, Ignasi Roig Quilis, Nuria Camiña, Ramón Roca Puig, Remei Blanco Guerrero, Marc Campayo

**Affiliations:** 1 Medical Oncology, Consorci Sanitari de Terrassa - Hospital Universitari, Terrassa, ESP; 2 Gynecology, Consorci Sanitari de Terrassa - Hospital Universitari, Terrassa, ESP; 3 Radiation Oncology, Consorci Sanitari de Terrassa - Hospital Universitari, Terrassa, ESP; 4 Pathology, Consorci Sanitari de Terrassa - Hospital Universitari, Terrassa, ESP; 5 Research and Innovation, Consorci Sanitari de Terrassa - Hospital Universitari, Terrassa, ESP

**Keywords:** metastatic breast cancer, oligometastatic, primary tumor, progression free-survival, radiotherapy, surgery

## Abstract

Background: Metastatic disease is the cause of death in most patients with breast cancer. The potential benefits of breast surgery (i.e., mastectomy or lumpectomy) of the primary tumour in patients with metastatic breast cancer are controversial. In clinical practice, selected multimetastatic and oligometastatic patients who show a complete metabolic response to systemic treatment receive local ablative treatment (breast surgery and/or radiotherapy) of their primary tumour.

Objectives: We described the local ablative treatment of primary tumours received by patients with oligometastatic and multimetastatic breast cancer with good systemic treatment response and analysed our cohort’s progression-free survival (PFS).

Patients and methods: A retrospective, descriptive cohort study was conducted at the Consorci Sanitari de Terrassa, Spain, from March 2013 to November 2023. We included all consecutive patients aged ≥18 years with histologically confirmed metastatic breast cancer who underwent local ablative treatment after receiving systemic therapy. Oligometastatic patients presented with up to five metastatic lesions.

Results: Seventeen patients were included in our study, 16 females and one male, with a median age of 50 years (range: 26-72). Most had luminal breast cancer (9, 53%) and were classified as oligometastatic (12, 71%). Their systemic treatments followed international guidelines for each molecular subtype. The most frequently performed local ablative treatment was mastectomy with axillary lymphadenectomy (7, 41%); eight patients (47%) received local ablative treatment (radiotherapy) for metastatic lesions. The median PFS from the date of diagnosis was not reached; at 60 months, the probability of not relapsing was 92%. The median PFS from the date of local ablative treatment was also not reached; at 60 months, the probability of not relapsing was 78%.

Conclusions: In this small local series, patients with oligometastatic or multimetastatic breast cancer achieving a good response to systemic therapy and receiving local ablative treatment of their primary tumour showed excellent PFS from the date of diagnosis and from the date of local ablative treatment.

## Introduction

Breast cancer is the most commonly diagnosed cancer in women worldwide, with more than 2.3 million new cases identified in 2022, and it is also the most common cause of cancer-related deaths in women [1]. In the European Union, 390,675 new cases were diagnosed in 2022, and 431,334 are estimated to be diagnosed in 2040 [2]. The five-year relative survival of women diagnosed with breast cancer in Europe is 82%, ranging from 74% in Eastern Europe to 85% in Northern Europe [3]. Although breast cancer-related deaths have been decreasing in developed countries, metastatic disease remains the underlying cause of death in most of these patients [4]. In this sense, a French study estimated that the overall survival of metastatic breast cancer patients was 37.22 months (95% confidence interval (CI): 36.3-38.04), with notable differences among the luminal (42.12 months, 95% CI: 40.90-43.10), HER2-positive (44.91 months, 95% CI: 42.51-47.90), and triple-negative (TN) (14.52 months, 95% CI: 13.70-15.24) subgroups [5]. In metastatic breast cancer, systemic therapy is initially recommended and, for patients showing good response, treating the primary tumour should be considered [6].

The standard therapy for metastatic breast cancer patients depends on the cancer's molecular subtype, varies between centres, and should be individualised according to patient tolerability [7]. However, some general trends can be established. In hormone receptor-positive/HER2-negative patients, the therapeutic approach consists of serial hormonal and targeted therapies until the disease becomes hormone-resistant; then, treatment should transition to chemotherapy or drugs being evaluated in clinical studies [7]. In HER2-positive patients, HER2-target agents combined with chemotherapy and hormonal therapy, if hormone receptor-positive, are advisable [7]. TN patients usually receive chemotherapy, and immunotherapy has been recently incorporated into the first-line regimens in some patients [7].

The potential benefits of breast surgery (i.e., mastectomy or lumpectomy) of the primary tumour in metastatic breast cancer patients remain controversial [8]. This procedure has been considered more appropriate in oligometastatic patients [8], although the definition of oligometastasis lacks consensus [9,10]. However, in clinical practice, selected multimetastatic patients who show a complete metabolic response to systemic treatment (leaving only residual locoregional disease) receive local ablative treatment (breast surgery and/or radiotherapy) of their primary tumour as well, albeit the evidence regarding this practice is non-existent.

Therefore, we evaluated the clinical outcomes of patients with stage IV breast cancer receiving local ablative treatment in our centre to improve our understanding of the benefits of this approach in this setting. We aimed to describe the baseline characteristics and treatments (initial systemic, local ablative, and adjuvant) of a group of patients with oligometastatic and multimetastatic (with good systemic treatment response) breast cancer. In addition, we analysed the progression-free survival (PFS) of our cohort from the date of diagnosis and from the date of local ablative treatment.

## Materials and methods

Study design and setting

This retrospective, descriptive cohort study was carried out at the Consorci Sanitari de Terrassa (CST, Spain) from March 2013 to November 2023, with a follow-up of at least 14 months after local ablative treatment. The study was approved by the Clinical Research Ethics Committee of the CST, and we obtained written informed consent from all study participants.

Participants

We included all consecutive patients aged ≥18 years with histologically confirmed metastatic breast cancer who underwent local ablative treatment after receiving systemic therapy. The definition of oligometastatic cancer is still a matter of debate. We chose the definition endorsed by the European Society for Medical Oncology to classify patients as oligometastatic if presenting a maximum of five metastatic lesions, not necessarily located in the same site, and all potentially susceptible to local ablative treatment [11]. Patients were otherwise classified as multimetastatic. We classified patients as multimetastatic when they presented metastatic disease (including patients with a high metastatic tumour burden) after receiving systemic treatment. They showed a complete metabolic response of distant lesions by PET/CT, but locoregional disease persisted.

Immunohistochemistry

We performed immunostaining for ERb on 4 µm sections of formalin-fixed, paraffin-embedded (FFPE) samples using the HercepTest™ mAb pharmDx (Dako Omnis) immunostaining system. We carried out antigen retrieval by immersing the preparations in Target Retrieval Solution Low pH for 30 minutes at 97 °C with the mouse monoclonal antibody clone DG44 RTU, incubating it for 10 minutes. We conducted immunohistochemistry for hormone receptors and Ki-67 on FFPE samples, starting with a 3 µm section stained with haematoxylin and eosin to verify the presence of an adequate number of invasive tumour cells. We prepared 4 µm serial sections and mounted them on adhesive slides specifically for immunostaining oestrogen receptor (ER), progesterone receptor (PR), and Ki-67. We performed all immunostaining using the Dako OMNIS equipment with the Envision Flex (Dako Omnis) visualisation system, and we immersed the preparations in Target Retrieval Solution for antigen retrieval. For ER immunostaining, we used the EP1 clone; for PR, the PgR 1294 clone from Dako; and for Ki-67, the MIB-1 clone from Dako.

According to the immunohistochemistry evaluation, we considered tumours positive for ER and PR when at least 1% of the tumour cells showed unequivocal nuclear staining, according to American Society of Clinical Oncology/College of American Pathologists (ASCO/CAP) guidelines [12]. HER2 was scored according to the pattern of membranous staining and percentage of stained malignant cells (0, no staining or faint incomplete staining in <10% of cells; 1, faint incomplete staining in >10% of cells; 2, weak to moderate complete staining in >10% of cells; and 3, strong complete staining in >10% of cells). Scores 2 (with FISH (fluorescence in situ hybridisation) amplification) and 3 were considered positive. Regarding Ki-67, the fraction of proliferating cells was based on a count of at least 500 tumour cells. Ki-67 values were expressed as the percentage of positive cells in each case. Cases with >20% positive nuclei were classified as high Ki-67 expression, and those with ≤20% were classified as low Ki-67 expression [13].

Intervention

Our patients formed a heterogeneous group and presented with varying metastatic patterns and histological subtypes. Therefore, distinct systemic treatment approaches were required, following the recommendations of scientific societies at the different times our patients were diagnosed over the years [11,14-16].

The local ablative treatment of the primary tumour received by patients included mastectomy, axillary lymphadenectomy, lumpectomy, selective lymph node biopsy, or any combination. Non-surgical radiotherapy radical treatment of the residual locoregional disease was also considered a local ablative treatment. The ablative treatment of metastases was always radiotherapy. For the breast, we used hypofractionated doses of 40.05 Gy at 2.67 Gy/s using the three-dimensional conformal radiation therapy technique; in cases of conservative surgery, a boost was also performed. For oligometastatic lesions, stereotactic body radiotherapy (SBRT) was performed using the volumetric modulated arc therapy technique, except for cases that did not meet SBRT criteria and were treated with the conventional technique.

Outcomes and measures

We gathered participants’ sociodemographic and clinicopathological variables before receiving local ablative treatment, including tumour size, nodal involvement, number of metastases, molecular subtype, and treatment received.

We analysed patients with luminal (ER- and/or PR-positive and HER2-negative), HER2 (ER- and PR-negative and HER2-positive), and TN (ER- and PR-negative and HER2-negative) breast cancers separately [17]. Because of our small sample size, we decided to merge into one group all patients with luminal histology, regardless of the luminal A (Ki-67 <20%) and luminal B (Ki-67 ≥20%) subtypes. We collected data on the type of local ablative treatment received, the need for adjuvant treatments, and whether the patient received treatment for the metastasis. We decided to analyse PFS in two ways: as the time between the date of the initial diagnosis and the date of the first progression or relapse, and as the time between the date of local ablative treatment and the date of disease progression.

Statistical analysis

We used absolute frequencies and percentages to describe categorical variables, and median and range for quantitative variables. We assessed PFS using the Kaplan-Meier product-limit method and compared distributions of the survival curves using log-rank tests. We used multivariate analyses (Cox regression model) to test the following variables for their impact on progression: tumour size (greater or less than 5 cm), nodal status (positive or negative), site of metastasis (nodal or bone versus visceral), molecular type of the tumour, first treatment received (including or excluding anthracyclines, and chemotherapy versus targeted treatment), radiological response of metastases to initial systemic treatment (according to RECIST (Response Evaluation Criteria in Solid Tumors) Criteria v.1.1), type of local ablative treatment of the primary tumour (surgery versus radiotherapy), type of surgery, and whether or not local ablative treatment of residual metastatic disease was performed. We calculated two-sided P-values and set the statistical significance level at P ≤ 0.05.

## Results

Baseline characteristics

A total of 17 patients were included in our study, 16 females and one male, with a median age of 50 years (range: 26-72 years). Most patients had luminal breast cancer (9, 53%), followed by HER2 (6, 35%) and TN (2, 12%). The majority, in all groups, presented with nodal involvement, whereas hormone sensitivity varied between groups (Table 1).

**Table 1:** Demographic and clinical characteristics of study participants according to tumour molecular type TN: Triple-negative.

Variables	Overall (n = 17)	Luminal (n = 9)	HER2 (n = 6)	TN (n = 2)
Sex, n (%)				
Female	16 (94)	8 (89)	6 (100)	2 (100)
Male	1 (6)	1 (11)	0 (0)	0 (0)
Age at diagnosis (years), median (range)	50 (26-72)	50 (26-72)	52 (34-64)	50 (47-53)
Race, n (%)				
Caucasian	17 (100)	9 (100)	6 (100)	2 (100)
Tumour size, n (%)				
≤5 cm (T1 and T2)	10 (59)	5 (56)	4 (67)	1 (50)
>5 cm (T3 and T4)	7 (41)	4 (44)	2 (33)	1 (50)
Nodal involvement, n (%)				
N0	4 (24)	3 (33)	1 (17)	0 (0)
N1-N3	13 (76)	6 (67)	5 (83)	2 (100)
Oestrogen receptor, n (%)				
Positive ≥1%	11 (65)	9 (100)	2 (33)	0 (0)
Negative <1%	6 (35)	0 (0)	4 (67)	2 (100)
Progesterone receptor, n (%)				
Positive ≥1%	10 (59)	8 (89)	2 (33)	0 (0)
Negative <1%	7 (41)	1 (11)	4 (67)	2 (100)
Ki-67 expression, n (%)				
High (>20%)	13 (76)	5 (56)	6 (100)	2 (100)
Low (≤20%)	4 (24)	4 (44)	0 (0)	0 (0)
Type of metastatic disease, n (%)				
Oligometastatic	12 (71)	7 (78)	3 (50)	2 (100)
Multimetastatic	5 (29)	2 (22)	3 (50)	0 (0)

When considering the metastatic status of study participants, most were classified as oligometastatic (12, 71%) and the remaining as multimetastatic (5, 29%). Nodal involvement was present in all multimetastatic patients (5, 100%) and in most oligometastatic patients (8, 67%). Again, hormone sensitivity varied between groups (Table 2).

**Table 2:** Demographic and clinical characteristics of study participants according to metastatic status

Variables	Overall (n = 17)	Oligometastatic (n = 12)	Multimetastatic (n = 5)
Sex, n (%)			
Female	16 (94)	11 (92)	5 (100)
Male	1 (6)	1 (8)	0 (0)
Age at diagnosis (years), median (range)	50 (26-72)	50 (26-72)	58 (34-60)
Race, n (%)			
Caucasian	17 (100)	12 (100)	5 (100)
Tumour size, n (%)			
≤5 cm (T1 and T2)	10 (59)	8 (67)	2 (40)
>5 cm (T3 and T4)	7 (41)	4 (33)	3 (60)
Nodal involvement, n (%)			
N0	4 (24)	4 (33)	0 (0)
N1-N3	13 (76)	8 (67)	5 (100)
HER2 overexpression, n (%)			
Yes	6 (35)	3 (25)	3 (60)
No	11 (65)	9 (75)	2 (40)
Oestrogen receptor, n (%)			
Positive ≥1%	11 (65)	9 (75)	2 (40)
Negative <1%	6 (35)	3 (25)	3 (60)
Progesterone receptor, n (%)			
Positive ≥1%	10 (59)	8 (67)	2 (40)
Negative <1%	7 (41)	4 (33)	3 (60)
Ki-67 expression, n (%)			
High (>20%)	13 (76)	8 (67)	4 (80)
Low (≤20%)	4 (24)	4 (33)	1 (20)

Treatment

The initial systemic treatment of patients was mostly chosen according to their molecular subtype: hormone therapy alone or in combination for luminal cancers, and anti-HER2 therapy for HER2-positive cancers. In two patients (12%), no surgery was performed on the primary tumour; instead, radical radiotherapy was administered to the locoregional residual disease following systemic treatment (Table 3).

**Table 3:** Treatment administered to study participants by breast cancer type, n (%) CDK4/6-i: CDK4/6 inhibitors; EC: epirubicin and cyclophosphamide; HT: hormone therapy; RT: radiotherapy; SLNB: selective lymph node biopsy; TN: triple-negative.

Variables	Overall (n = 17)	Luminal (n = 9)	HER2 (n = 6)	TN (n = 2)
Initial systemic treatment				
EC + Taxanes	4 (24)	3 (33)	0 (0)	1 (50)
EC + Taxanes + Carboplatin	1 (6)	0 (0)	0 (0)	1 (50)
EC + Taxanes + Anti-HER2	2 (12)	0 (0)	2 (33)	0 (0)
Taxanes + Anti-HER2	4 (24)	0 (0)	4 (67)	0 (0)
HT	1 (6)	1 (11)	0 (0)	0 (0)
HT + CDK4/6-i	5 (29)	5 (56)	0 (0)	0 (0)
Local ablative treatment				
Primary				
Tumourectomy	2 (12)	1 (11)	1 (17)	0 (0)
Tumourectomy + SLNB	2 (12)	1 (11)	1 (17)	0 (0)
Tumourectomy + Lymphadenectomy	3 (18)	1 (11)	1 (17)	1 (50)
Mastectomy + SLNB	1 (6)	1 (11)	0 (0)	0 (0)
Mastectomy + Lymphadenectomy	7 (41)	4 (44)	2 (33)	1 (50)
RT	2 (12)	1 (11)	1 (17)	0 (0)
Metastasis				
None	9 (53)	4 (44)	5 (83)	0 (0)
RT bone M1	5 (29)	5 (56)	0 (0)	0 (0)
RT nodal M1	2 (12)	0 (0)	1 (17)	1 (50)
RT nodal and bone M1	1 (6)	0 (0)	0 (0)	1 (50)
Adjuvant treatment				
RT	6 (35)	3 (33)	1 (17)	2 (100)
RT + Anti-HER2	1 (6)	0 (0)	1 (17)	0 (0)
RT + HT	2 (12)	2 (22)	0 (0)	0 (0)
HT	2 (12)	1 (11)	1 (17)	0 (0)
RT + Anti-HER2 + HT	1 (6)	0 (0)	1 (17)	0 (0)
None	5 (29)	3 (33)	2 (33)	0 (0)

Oligometastatic patients generally received combinations of chemotherapy with anthracyclines and taxanes (4, 33%), with the addition of anti-HER2 monoclonal antibodies when overexpression of this receptor was detected (3, 25%). For most patients with metastatic disease, the initial treatment regimen consisted of taxanes and anti-HER2 monoclonal antibodies (3, 60%) and hormone therapy alone or in combination for the two patients (40%) with hormone receptor-positive tumours (Table 4).

**Table 4:** Treatment administered to study participants by type of metastasis, n (%) CDK4/6-i: CDK4/6 inhibitors; EC: epirubicin and cyclophosphamide; HT: hormone therapy; RT: radiotherapy; SLNB: selective lymph node biopsy.

Variables	Overall (n = 17)	Oligometastatic (n = 12)	Multimetastatic (n = 5)
Initial systemic treatment			
EC + Taxanes	4 (24)	4 (33)	0 (0)
EC + Taxanes + Carboplatin	1 (6)	1 (8)	0 (0)
EC + Taxanes + Anti-HER2	2 (12)	2 (17)	0 (0)
Taxanes + Anti-HER2	4 (24)	1 (8)	3 (60)
HT	1 (6)	0 (0)	1 (20)
HT + CDK4/6-i	5 (29)	4 (33)	1 (20)
Local ablative treatment			
Primary			
Tumourectomy	2 (12)	0 (0)	2 (40)
Tumourectomy + SLNB	2 (12)	2 (17)	0 (0)
Tumourectomy + Lymphadenectomy	3 (18)	3 (25)	0 (0)
Mastectomy + SLNB	1 (6)	1 (8)	0 (0)
Mastectomy + Lymphadenectomy	7 (41)	5 (42)	2 (40)
RT	2 (12)	1 (8)	1 (20)
Metastatic			
None	9 (53)	5 (42)	4 (80)
RT bone M1	5 (29)	5 (42)	0 (0)
RT nodal M1	2 (12)	1 (8)	1 (20)
RT nodal and bone M1	1 (6)	1 (8)	0 (0)
Adjuvant treatment			
RT	6 (35)	5 (42)	1 (20)
RT + Anti-HER2	1 (6)	0 (0)	1 (20)
RT + HT	2 (12)	2 (17)	0 (0)
HT	2 (12)	2 (17)	0 (0)
RT + Anti-HER2 + HT	1 (6)	1 (8)	0 (0)
None	5 (29)	2 (17)	3 (60)

Overall, the most frequently performed local ablative treatment was mastectomy with axillary lymphadenectomy (7, 41%), and eight patients (47%) received local ablative treatment (radiotherapy) for metastatic lesions (always nodal or bone metastases). A total of 12 patients (71%) received some form of adjuvant treatment, most commonly radiotherapy, and systemic treatment was added based on their molecular profile (Tables 3, 4).

Progression-free survival analysis

**Figure 1:**
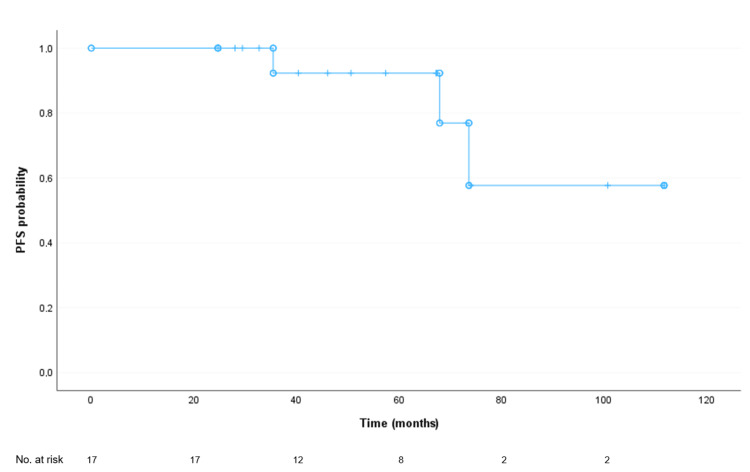
Progression-free survival (PFS) of study participants from breast cancer diagnosis Median PFS: Not reached. Mean PFS (limited to the longest survival time, if censored): 91.8 months (95% confidence interval: 73.9-109.7 months.

**Figure 2:**
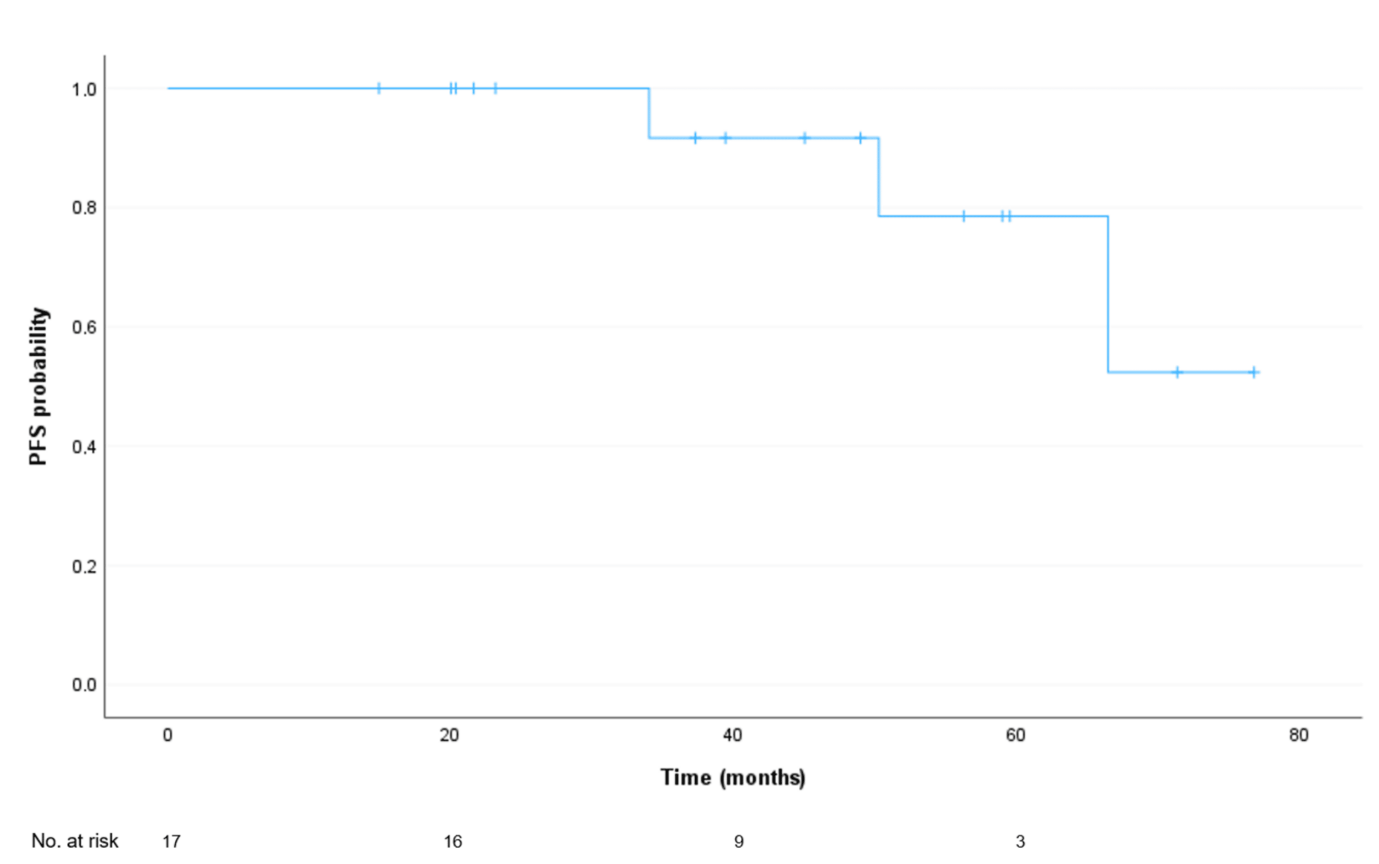
Progression-free survival (PFS) of study participants from intervention or local ablative treatment Median PFS: Not reached. Mean PFS (limited to the longest survival time, if censored): 67.1 months (95% confidence interval: 58.1-76.0 months).

The median PFS from the date of diagnosis was not reached, with an estimated mean follow-up of 91.8 months. At 60 months of follow-up, there was a 92% probability of not relapsing (Figure 1). The median PFS from the date of local ablative treatment was also not reached, with an estimated mean follow-up of 67.05 months. At 60 months of follow-up after this treatment, there was a 78% probability of not relapsing (Figure 2). None of the clinicopathological factors analysed showed a correlation with PFS (data not shown).

## Discussion

Our 17 patients formed a small and heterogeneous cohort with metastatic breast cancer, 12 of whom were oligometastatic and five had multimetastatic disease. The histological types were also varied, and their initial systemic treatment depended on their molecular subtype. The most common local ablative treatment was mastectomy with lymphadenectomy. Almost half of our cohort received radiotherapy for metastatic lesions, and most were irradiated as part of their adjuvant treatment. Neither the median PFS from the date of diagnosis nor from the date of local ablative treatment was reached.

In our study, nearly all patients were progression-free five years after receiving their first treatment for metastatic breast cancer. This result exceeded the expected PFS in the general population of patients with metastatic breast cancer [5,18-20] and is consistent with the findings of other retrospective series on oligometastatic breast cancer [21-23]. However, these observations should be interpreted with caution. The oligometastatic condition has been hypothesised to carry a better prognosis, as it reflects less aggressive tumour biology (more cellular adhesion, less mobility, reduced survival in the blood or lymphatic stream, and a diminished capacity to proliferate in secondary tissues) [24]. Therefore, the value of a radical approach to treating metastatic lesions and the primary tumour should be established through randomised prospective studies, which are very limited in this context.

Turning to randomised trials, the MF07-01 [25], ABCSG-POSITIVE [26], ECOG-ACRIN 2108 [27], Tata Memorial Centre [28], and NRG-BR002 [29] studies did not demonstrate an increase in overall survival for patients who underwent local ablative treatment. However, the first study did show a trend towards achieving this goal with a longer follow-up than initially planned. These studies suffered from significant heterogeneity in terms of the molecular subtypes of the included patients, the systemic treatments administered, and whether ablative treatments were performed on metastases (in oligometastatic patients) beyond the radical treatment of the primary tumour. Given everything mentioned above, drawing conclusions about the potential benefit of ablative treatment of the primary tumour in patients with metastatic breast cancer is complex. However, a single study seemed to detect a certain benefit in patients with the luminal phenotype [25].

Due to the small size of our series, we did not expect to obtain significant results to emphasise the use of local ablative treatment in a specific group of patients defined by clinicopathological factors. For the same reason, we did not consider it appropriate to analyse overall survival results. Nonetheless, we considered it important to present our findings, since our impression was that, overall, our patients benefited from local ablative treatments of their primary tumours, as demonstrated by their high PFS values. Notably, previous reports did not consider the response to systemic treatment a criterion for considering local ablative treatment and, contrary to our study, did not include patients with multimetastasis showing a complete metabolic response, leaving only locoregional residual disease. In our cohort, these patients represented 29% of those analysed (among whom 60% were HER2-positive) and, to the best of our knowledge, they have not been specifically evaluated before. This patient selection may explain the difference between our data and the results of the studies mentioned above [25-29]. This suggests that perhaps not only the magnitude of metastatic disease, but also tumour biology and the response to systemic treatment, could be determining factors in considering local treatment for oligometastatic and multimetastatic patients.

However, our results should be interpreted in light of the study’s limitations, mainly stemming from its descriptive and retrospective nature and, therefore, the absence of a control group. In addition, the small sample size could preclude extrapolation of our patients’ outcomes to larger populations. The short minimum follow-up and the heterogeneity of our sample should also be taken into account when evaluating our findings.

## Conclusions

Treatments received by patients with oligometastatic or multimetastatic (with good systemic treatment response) breast cancer who underwent local ablative treatment of their primary tumour depended on the molecular subtype. The analysis of our cohort’s follow-up showed excellent PFS from the date of diagnosis and from the date of local ablative treatment. Although highly limited, we considered that our results were sufficiently compelling to encourage further research in this area. Specifically, we call for prospective, randomised studies that take into account the different molecular subtypes and the patient’s response to systemic treatments.

## References

[1] Bray F, Laversanne M, Sung H, Ferlay J, Siegel RL, Soerjomataram I, Jemal A (2024). Global cancer statistics 2022: GLOBOCAN estimates of incidence and mortality worldwide for 36 cancers in 185 countries. CA Cancer J Clin.

[2] (2024). ECIS - European cancer information system. https://ecis.jrc.ec.europa.eu/.

[3] Sant M, Chirlaque Lopez MD, Agresti R (2015). Survival of women with cancers of breast and genital organs in Europe 1999-2007: results of the EUROCARE-5 study. Eur J Cancer.

[4] Redig AJ, McAllister SS (2013). Breast cancer as a systemic disease: a view of metastasis. J Intern Med.

[5] Gobbini E, Ezzalfani M, Dieras V (2018). Time trends of overall survival among metastatic breast cancer patients in the real-life ESME cohort. Eur J Cancer.

[6] Liu B, Liu H, Liu M (2023). Aggressive local therapy for de novo metastatic breast cancer: challenges and updates (Review). Oncol Rep.

[7] Waks AG, Winer EP (2019). Breast cancer treatment: a review. JAMA.

[8] Tosello G, Torloni MR, Mota BS, Neeman T, Riera R (2018). Breast surgery for metastatic breast cancer. Cochrane Database Syst Rev.

[9] Kwapisz D (2019). Oligometastatic breast cancer. Breast Cancer.

[10] Kent CL, McDuff SG, Salama JK (2021). Oligometastatic breast cancer: where are we now and where are we headed? A narrative review. Ann Palliat Med.

[11] Gennari A, André F, Barrios CH (2021). ESMO clinical practice guideline for the diagnosis, staging and treatment of patients with metastatic breast cancer. Ann Oncol.

[12] Hammond ME, Hayes DF, Dowsett M (2010). American Society of Clinical Oncology/College of American Pathologists guideline recommendations for immunohistochemical testing of estrogen and progesterone receptors in breast cancer. J Clin Oncol.

[13] Klöppel G, Couvelard A, Perren A (2009). ENETS consensus guidelines for the standards of care in neuroendocrine tumors: towards a standardized approach to the diagnosis of gastroenteropancreatic neuroendocrine tumors and their prognostic stratification. Neuroendocrinology.

[14] Senkus E, Kyriakides S, Penault-Llorca F, Poortmans P, Thompson A, Zackrisson S, Cardoso F (2013). Primary breast cancer: ESMO clinical practice guidelines for diagnosis, treatment and follow-up. Ann Oncol.

[15] Coates AS, Winer EP, Goldhirsch A (2015). Tailoring therapies--improving the management of early breast cancer: St Gallen international expert consensus on the primary therapy of early breast cancer 2015. Ann Oncol.

[16] Cardoso F, Costa A, Senkus E (2017). 3rd ESO-ESMO international consensus guidelines for advanced breast cancer (ABC 3). Breast.

[17] Goldhirsch A, Winer EP, Coates AS, Gelber RD, Piccart-Gebhart M, Thürlimann B, Senn HJ (2013). Personalizing the treatment of women with early breast cancer: highlights of the St Gallen international expert consensus on the primary therapy of early breast cancer 2013. Ann Oncol.

[18] Bartlett CH, Mardekian J, Cotter MJ, Huang X, Zhang Z, Parrinello CM, Bourla AB (2020). Concordance of real-world versus conventional progression-free survival from a phase 3 trial of endocrine therapy as first-line treatment for metastatic breast cancer. PLoS One.

[19] Saleh K, Carton M, Dieras V (2021). Impact of body mass index on overall survival in patients with metastatic breast cancer. Breast.

[20] Miglietta F, Bottosso M, Griguolo G, Dieci MV, Guarneri V (2022). Major advancements in metastatic breast cancer treatment: when expanding options means prolonging survival. ESMO Open.

[21] Kobayashi T, Ichiba T, Sakuyama T (2012). Possible clinical cure of metastatic breast cancer: lessons from our 30-year experience with oligometastatic breast cancer patients and literature review. Breast Cancer.

[22] Nagasaki E, Kudo R, Tamura M (2021). Long-term outcomes of oligometastatic breast cancer patients treated with curative intent: an updated report. Breast Cancer.

[23] Ueno T, Bi X, Liu G (2020). International retrospective cohort study of locoregional and systemic therapy in oligometastatic breast cancer (OLIGO-BC1). J Clin Oncol.

[24] AlGhamdi H, Dhont J, Krayem M, De Bruyn P, Engels B, Van Gestel D, Van den Begin R (2022). The road to dissemination: the concept of oligometastases and the barriers for widespread disease. Cancers (Basel).

[25] Soran A, Ozmen V, Ozbas S (2018). Randomized trial comparing resection of primary tumor with no surgery in stage IV breast cancer at presentation: protocol MF07-01. Ann Surg Oncol.

[26] Fitzal F, Bjelic-Radisic V, Knauer M (2019). Impact of breast surgery in primary metastasized breast cancer: outcomes of the prospective randomized phase III ABCSG-28 POSYTIVE trial. Ann Surg.

[27] Khan SA, Zhao F, Goldstein LJ (2022). Early local therapy for the primary site in de novo stage IV breast cancer: results of a randomized clinical trial (EA2108). J Clin Oncol.

[28] Badwe R, Hawaldar R, Nair N (2015). Locoregional treatment versus no treatment of the primary tumour in metastatic breast cancer: an open-label randomised controlled trial. Lancet Oncol.

[29] Chmura SJ, Winter KA, Woodward WA (2022). NRG-BR002: a phase IIR/III trial of standard of care systemic therapy with or without stereotactic body radiotherapy (SBRT) and/or surgical resection (SR) for newly oligometastatic breast cancer (NCT02364557). J Clin Oncol.

